# A Review of Medical Image Registration for Different Modalities

**DOI:** 10.3390/bioengineering11080786

**Published:** 2024-08-02

**Authors:** Fatemehzahra Darzi, Thomas Bocklitz

**Affiliations:** 1 Institute of Physical Chemistry, Friedrich Schiller University Jena, Helmholtzweg 4, 07743 Jena, Germany; fatemehzahra.darzi@uni-jena.de; 2 Department of Photonic Data Science, Leibniz Institute of Photonic Technology, Albert-Einstein-Straße 9, 07745 Jena, Germany

**Keywords:** image registration, multimodal, unimodal, medical images, alignment, image processing

## Abstract

Medical image registration has become pivotal in recent years with the integration of various imaging modalities like X-ray, ultrasound, MRI, and CT scans, enabling comprehensive analysis and diagnosis of biological structures. This paper provides a comprehensive review of registration techniques for medical images, with an in-depth focus on 2D-2D image registration methods. While 3D registration is briefly touched upon, the primary emphasis remains on 2D techniques and their applications. This review covers registration techniques for diverse modalities, including unimodal, multimodal, interpatient, and intra-patient. The paper explores the challenges encountered in medical image registration, including geometric distortion, differences in image properties, outliers, and optimization convergence, and discusses their impact on registration accuracy and reliability. Strategies for addressing these challenges are highlighted, emphasizing the need for continual innovation and refinement of techniques to enhance the accuracy and reliability of medical image registration systems. The paper concludes by emphasizing the importance of accurate medical image registration in improving diagnosis.

## 1. Introduction

In recent years, medical image registration has developed as a critical area of research due to the integration of various imaging modalities like X-ray, ultrasound, MRI, and CT scans. This integration has significantly advanced the field, enabling comprehensive analysis and diagnosis of biological structures. Image registration plays a crucial role in this advancement, allowing for the alignment of images to standardized reference frames. It involves the use of various transformation models, similarity measures, and optimization techniques [1]. Deep learning has been increasingly applied in this field, with algorithms such as Voxelmorph, Demons, and SyN being used for medical image registration [2]. The process involves feature detection, matching, transform model estimation, and image resampling and transformation. These methods have significant potential to improve image visualization for different human anatomy in medical diagnosis and treatment. This paper presents a comprehensive review of registration techniques for biological medical images obtained from diverse modalities, including unimodal, multimodal, interpatient, and intra-patient [3].

**Figure 1:**
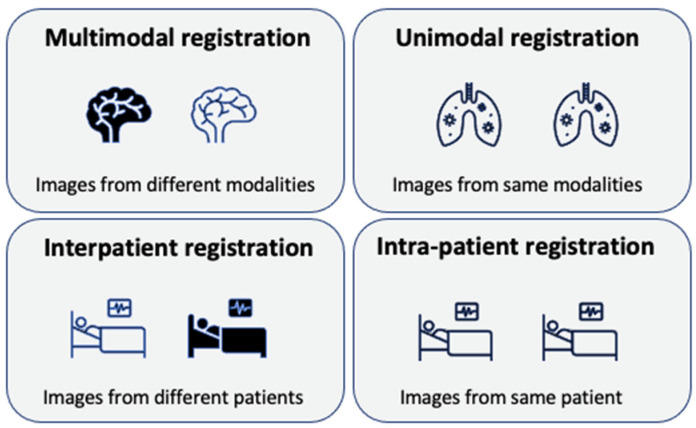
Illustration of different types of image registration in medical imaging. The figure displays four quadrants representing various registration scenarios. The top-left quadrant depicts multimodal image registration, aligning images acquired from different sensors or modalities. The top-right quadrant shows unimodal registration, aligning images obtained from the same modality or sensor. The bottom-left quadrant illustrates interpatient registration, aligning images from different individuals. Finally, the bottom-right quadrant represents intra-patient registration, aligning images obtained from the same individual over time, either on the same day or on different days.

In this paper, we explore various types of image registration techniques in medical imaging. Unimodal registration, as depicted in Figure 1, refers to the process of aligning images obtained from the same type of imaging sensors or modalities, ensuring that they are spatially aligned. In unimodal registration, the goal is to align images captured using a single imaging modality to enable precise comparison and analysis. This technique is crucial in various applications, particularly in medical imaging, where it enables precise comparison and analysis of images acquired from the same imaging modality, such as multiple MRI scans or X-ray images. The importance of unimodal registration lies in its ability to correct spatial discrepancies and align anatomical structures or features within images, facilitating detailed analysis and informed decision-making. It finds extensive use in longitudinal studies for tracking disease progression, motion correction in dynamic imaging, atlas-based segmentation, image quality enhancement through averaging, intra-operative guidance, quality control processes, and change detection in fields like remote sensing. The primary advantage of unimodal registration is its relative simplicity compared to multimodal approaches, as images from the same modality typically share similar intensity characteristics and feature representations. This similarity often allows for more straightforward and robust registration processes, employing simpler similarity metrics and transformation models. However, challenges such as intensity inhomogeneities, noise, or anatomical variations between scans still necessitate the careful selection of registration algorithms and parameters to achieve optimal results in unimodal registration tasks [4].

In contrast, multimodal image registration involves aligning images of the same scene acquired from different sensors, at different times, or from varying perspectives, such as the registration of MRI and X-ray images. If images of the same modality and different measurement parameters are registered in some cases, a multimodal image registration task also evolves. For example, when considering MRI scans acquired using different pulse sequences or scan protocols, we classify them as either multimodal or unimodal. Different pulse sequences produce completely different images due to variations in tissue contrast, spatial resolution, and other parameters. If these differences are substantial and significantly impact the information content, we consider the scans as multimodal. However, when the differences are minimal, they can be categorized as unimodal. As we know, X-ray images can suffer from geometric distortions due to variations in the angle of X-ray beam projection and the position of the patient. Additionally, X-ray images are prone to artifacts such as scatter radiation, which can obscure details and reduce the image quality. MRI images are also subject to several types of distortions, including geometric distortions caused by magnetic field inhomogeneities and susceptibility artifacts [5]. These distortions can result in spatial inaccuracies, particularly near interfaces of different tissue types or near metallic implants. Furthermore, patient movement during the scan can introduce motion artifacts, which can blur the images and complicate an accurate interpretation. These distortions can lead to misalignment and inaccuracies in the representation of anatomical structures. Multimodal co-registration techniques are employed to mitigate these distortions and improve the accuracy of image alignment. By aligning images from different modalities, co-registration can correct spatial discrepancies and enhance the reliability of combined image analysis. This process is essential for ensuring precise and reliable interpretation, which is particularly important in clinical decision-making and educational settings. This registration process aims to geometrically align these images to enable comparison, fusion, or analysis. Multimodal image registration methods can be categorized into feature-based, where distinct features are extracted and matched, and area-based, which directly processes image intensity values without identifying specific features [6].

Interpatient registration refers to the process of aligning images obtained from different patients. This type of registration can be challenging due to the differences in underlying anatomical structures across individuals. Accurate interpatient registration is essential for tasks that involve comparing images or data from different patients, such as in research studies or treatment planning where a standard frame of reference is needed. Intra-patient registration, on the other hand, involves aligning images acquired from the same patient, either on the same day (same-day intra-patient) or on different days (different-day intra-patient). This type of registration can be challenging due to changes in the image appearance caused by factors like metabolic processes, weight fluctuations, or bowel movements. Intra-patient registration is crucial for applications such as monitoring disease progression, assessing treatment outcomes, or performing pre- and post-procedural image comparisons within the same individual over time [7].

While this review primarily focuses on 2D image registration techniques, it is important to acknowledge the role of registration aids such as fiducial markers in image alignment across various modalities. Fiducials, which are external reference points or markers placed on or near the subject being imaged, significantly facilitate the registration process by providing consistent reference points across different images [7]. These markers prove particularly useful in scenarios where anatomical landmarks may be unclear or inconsistent. Fiducial-based registration, while extensively used in 3D datasets, also has significant applications in 2D image registration, especially in cases requiring high-precision alignment [8]. In 2D medical imaging, fiducial markers can be external (attached to the patient’s skin) or internal (implanted or anatomical landmarks). Common techniques in 2D fiducial-based registration include point-based methods, where corresponding fiducial points are matched between images, and contour-based methods, which align fiducial shapes or outlines. These methods often employ least squares fitting algorithms to minimize the distance between corresponding fiducials. For instance, in radiotherapy planning, radio-opaque markers are frequently used to align treatment images with reference images, ensuring the accurate targeting of radiation beams [9]. Additionally, in surgical navigation, skin-affixed fiducials help register preoperative images to intraoperative patient positioning [10]. While fiducial-based methods offer high accuracy, they must be balanced against considerations such as invasiveness, marker stability, and the potential need for additional imaging procedures.

The challenges during image registration in the medical domain include obstacles such as the lack of standardized ground truth labels for evaluating the registration outcomes convincingly, subjective assessments by medical experts, instability in outcomes from various registration algorithms, and the influence of data quality variations on the registration results [4]. Additionally, issues such as geometric distortions and differences in image properties like intensity, color distributions, and resolution, as well as the impact of noise, can pose significant hurdles during the image registration process in the medical context. Addressing these challenges is crucial to enhancing the accuracy and reliability of medical image registration systems for improved clinical applications.

In the upcoming sections of this review, we will explore different image registration techniques, examining their properties and addressing the challenges inherent in their application across different contexts. Additionally, we will provide an overview of feature-based and area-based frameworks for image registration, highlighting the challenges of aligning images. We will delve into the general image registration framework, offering a comprehensive understanding of the registration process.

## 2. Materials and Methods

In this section, we explore the fundamental principles of general registration frameworks. Image registration, a critical component of image analysis, involves mapping two images on both the spatial and intensity levels.

Let us denote $\left(X_{Fix},\;Y_{Fix}\right)$ as the coordinates of points in the fixed image and $\left(X_{Mov},\;Y_{Mov}\right)$ as the (pixel, line) coordinates of the corresponding points in the moving image undergoing registration. The relationship between $\left(X_{Fix},\;Y_{Fix}\right)$ and $\left(X_{Mov},\;Y_{Mov}\right)$ can be expressed as follows:

$$X_{Fix}=f\left(X_{Mov},\;Y_{Mov}\right)\;Y_{Fix}=g\left(X_{Mov},\;Y_{Mov}\right)$$

where f and g represent the functions that accurately represent the relationship between the coordinates of the two images [11].

Within the realm of image registration, researchers systematically categorize methodologies into two primary groups: area-based and feature-based methods. Area-based methods are preferable when there are no prominent details in an image and the identifying feature is gray level/color rather than local shape. An example of an area-based method is correlation-based registration, which is extensively used for registering monomodal images, particularly in medical applications [12]. Conversely, feature-based matching methods become imperative when image intensities contain richer local structural information. These methods leverage feature extraction algorithms to discern and extract essential image features [13]. An example of a feature-based method is the scale invariant feature transform (SIFT) algorithm [14], which is widely used for its robustness in detecting and describing local features in images.

**Figure 2:**
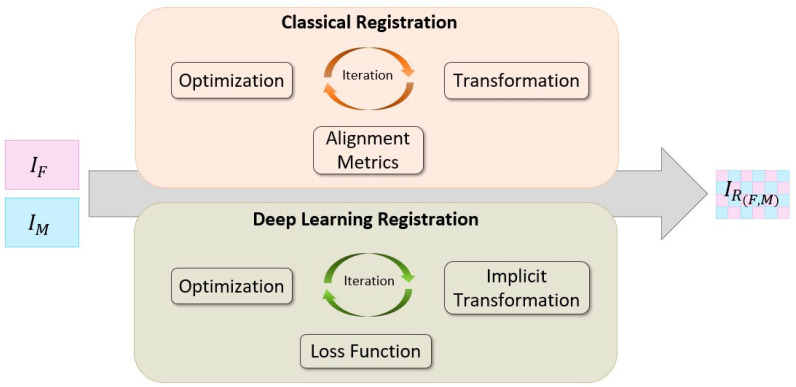
General framework of image registration. This figure presents a comprehensive overview of the image registration process, showcasing both classical and deep learning approaches. The framework includes two input images, a fixed image ($I_{F}$) and a moving image ($I_{M}$). In the classical registration area, alignment metrics, transformation, and optimization are depicted as key components, emphasizing their iterative interaction for achieving accurate alignment. Additionally, the deep learning registration section showcases the inclusion of a loss function, implicit transformation, and optimization within the iterative process.

As we progress into the broader context of image registration frameworks, it becomes evident that three pivotal components play an indispensable role: alignment metrics, transformation models, and optimization methods. Alignment metrics furnish a quantitative measure of image similarity, guiding the entire registration process. Transformation models define the spatial mapping between images, enabling alignment to occur seamlessly. Optimization methods diligently refine the transformation parameters based on selected alignment metrics, striving to minimize registration error and enhance alignment accuracy. Each step contributes significantly to the overall success of image registration, highlighting the delicate balance between accuracy and computational efficiency in this critical field of study. Figure 2 provides a comprehensive overview of the image registration process, illustrating both classical and deep learning approaches. In the following sections, we will delve into each of these components.

### 2.1. Alignment Metrics

Alignment metrics are fundamental in image registration, quantifying the similarity or dissimilarity between images to establish meaningful correspondence. These metrics serve dual purposes: evaluating the registration process and optimizing the registration parameters. Traditionally, hand-developed features guide registration but face limitations in matching diverse image data. Recent advancements have introduced learning-based methods that select features from a large pool to characterize each pixel [15].

In the context of image registration, alignment metrics utilize feature extraction techniques to detect distinctive key points like corners or blobs. Key point selection can be manual or automatic. Manual selection is time-consuming and prone to inaccuracies; hence, automatic feature selection algorithms without human intervention have been proposed. It is crucial for these algorithms to be applicable across different applications without being overly specific [16].

Among automatic feature detection methods, several algorithms have gained prominence, including binary robust invariant scalable key points (BRISK) [17], the scale invariant feature transform (SIFT) algorithm [14], features from accelerated segment test (FAST) [18], binary robust independent elementary features (BRIEF) [19], accelerated-KAZE (AKAZE) [20], and oriented FAST and rotated BRIEF (ORB) [21]. Notably, BRISK efficiently detects features with robust description, making it well suited for image registration tasks. AKAZE, an accelerated version of KAZE, excels in capturing both scale and space information, ensuring enhanced repeatability and robustness. The next algorithm, ORB, demonstrates speed and robustness, proving suitable for real-time applications, including image registration tasks. These feature detection methods, especially BRISK, AKAZE, and ORB, are widely used in computer vision, showcasing effectiveness across diverse applications and positioning them as valuable tools for accurate alignment and correspondence estimations in image registration tasks.

### 2.2. Transformation Models

In the realm of image registration, the primary goal is to align related images by establishing correspondences between their features and applying transformations. Transformations are essential tools in this process, as they enable the adjustment of one image to match the spatial configuration of another. The need for transformations arises from the inherent variability in images acquired at different times, angles, or using diverse devices. These variabilities may include differences in scale, orientation, and spatial position, making it imperative to apply transformations that can rectify such discrepancies.

**Figure 3:**
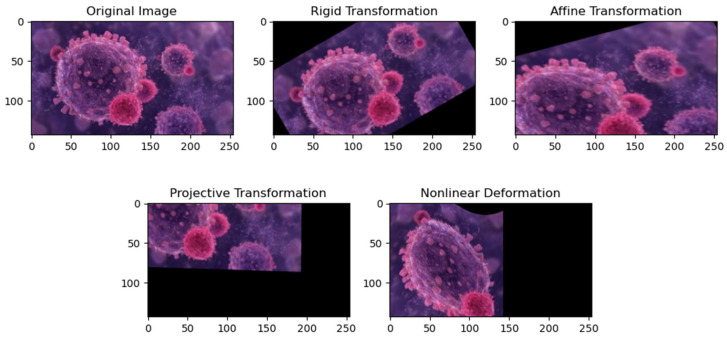
Different types of transformation. This figure illustrates various transformations applied to an original image. (1) Rigid transformation preserves distances and angles; (2) affine transformation incorporates translation, rotation, scaling, and shearing; (3) projective transformation accommodates perspective distortions; and (4) nonlinear deformation addresses intricate deformations. The original image is displayed alongside the transformed versions for comparison.

Geometric transformations, such as rigid, affine, projective, and nonlinear transformations, serve as mechanisms to bring images into spatial congruence. Rigid transformation is a fundamental technique characterized by its ability to preserve distances and angles. This transformation is particularly suitable for aligning images with minimal deformations, making it a valuable tool in scenarios where maintaining the structural integrity of the objects or subjects within the images is crucial. Common applications include aligning medical images for tasks such as tracking changes over time or facilitating a comparative analysis [22]. Affine transformation is a versatile method incorporating translation, rotation, scaling, and shearing. This flexibility allows for a broad range of adjustments during the alignment process. Affine transformations are well suited for applications where a more comprehensive adjustment is needed, such as aligning images with varying sizes, orientations, or spatial relationships. Medical imaging, computer vision, and remote sensing are areas where affine transformations find extensive use [23]. Projective transformation is essential for dealing with perspective distortions and more complex spatial changes in images. This transformation accommodates more complex spatial changes, making it valuable for applications where the scenes captured in images exhibit significant depth variations or non-uniform perspectives. Fields such as computer graphics, virtual reality, and architectural imaging often benefit from projective transformations to rectify distortions introduced by the viewing perspective [24]. Nonlinear deformations cater to intricate transformations beyond the capabilities of the rigid and affine methods, making them indispensable for scenarios involving complex anatomical changes. It allows for a more nuanced adjustment of the spatial relationships within the images, making it indispensable in tasks where subtle and intricate transformations are critical for accurate registration [25,26,27]. Figure 3 illustrates various transformations applied to an original image.

The choice of a particular spatial transformation depends on the characteristics of the images being registered and the specific requirements of the application. In certain cases, a combination of different transformation models may be necessary to achieve optimal registration results [28]. By employing these transformations, image registration achieves the crucial task of bringing images into a common spatial framework, facilitating meaningful comparisons, analyses, and interpretations in various domains, such as medical imaging, computer vision, and remote sensing.

### 2.3. Optimization Methods

Image registration involves solving an optimization problem to achieve accurate alignment between images. The objective is to minimize dissimilarity or maximize similarity measures by identifying the optimal set of transformation parameters [29].

Advanced optimization algorithms commonly employed in image registration include numerical Gauss–Newton minimization and gradient descent optimization. These methods iteratively update the transformation parameters by computing the gradient or the Hessian matrix of the dissimilarity measure. Navigating the parameter space by following the gradient direction or using a suitable step size allows these algorithms to converge towards the optimal solution. Another optimization method is the Levenberg–Marquardt algorithm, particularly useful for reducing pixel intensity variance in the registration process. By incorporating a regularization term and balancing between the Gauss–Newton and gradient descent steps, the Levenberg–Marquardt algorithm improves the stability and convergence of the optimization process [30,31,32].

**Figure 4:**
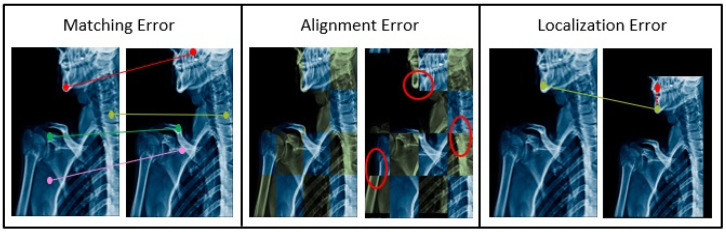
This figure illustrates three common types of errors encountered during image registration. In the left panel, a matching error is depicted where key points (denoted by colored markers) in the fixed image fail to align precisely with the corresponding key points in the moving image. This discrepancy underscores the challenge of achieving accurate correspondence between key points in image registration. In the center panel, an alignment error is observed. Here, the vertebral column exhibits misalignment when comparing images, as indicated by the highlighted deviation (red oval) from the expected anatomical alignment. Addressing such alignment discrepancies is crucial for ensuring precise image registration and clinical assessment. The right panel illustrates a localization error where the placement of a key point inaccurately corresponds to the targeted anatomical feature. This means that, while the key point is present in both the reference (fixed) and moving images, its position is shifted or misaligned relative to its intended anatomical location. Inaccuracies like these can significantly affect the precision of diagnostic evaluations and treatment planning. Such errors can significantly impact the diagnostic accuracy and subsequent treatment planning processes. Each type of error underscores distinct challenges in achieving precise and reliable image registration.

During optimization, various errors can occur, as shown in Figure 4, including [33]:

- Localization error: Arises when a key point is not accurately detected, causing their coordinates to shift. It can be minimized by using a good detection algorithm.
- Matching error: Measures the quality of key point detections by counting the number of false matches found between control point candidates.
- Alignment error: Arises when there are geometric distortions between images that deviate from the expected mapping model used for registration. This deviation can be quantified at the key point by calculating the mean squared error, providing a measure of the discrepancy between the actual and expected positions.

In conclusion, general image registration frameworks offer a systematic approach to aligning images through the consideration of alignment metrics, transformation models, and optimization methods. Ongoing research aims to enhance the adaptability, accuracy, and efficiency in image registration, contributing to the advancement of versatile and robust computer vision systems for applications such as image restoration, target tracking, image stitching, image fusion, 3D reconstruction, and pattern recognition. Addressing the challenges associated with image registration is crucial for pushing the boundaries of computer vision development.

## 3. Classification of Image Registration Techniques

**Figure 5:**
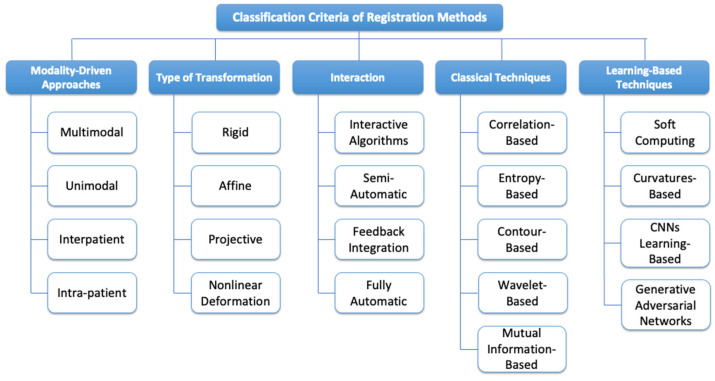
Classification of registration methods. This figure presents a comprehensive classification scheme for registration methods, highlighting five main subsets: modalities, type of transformation, interaction, classical techniques, and learning-based techniques. Each subset further encompasses various subcategories, providing a detailed taxonomy for organizing and understanding different registration approaches.

Image registration is both theoretically important and practically useful. There are a number of criteria that can be used to classify image registration techniques and methods. Figure 5 illustrates a classification of registration methods, presenting a comprehensive scheme that highlights five main subsets. These subsets offer a structured framework for categorizing and analyzing the diverse range of image registration techniques available. The modality-driven approaches and transformation methods, having been discussed in previous sections, will not be reiterated here. The remaining classification categories are as follows:

- Interaction [34]:
  1. Interactive: Involving active user participation, interactive registration allows users to guide the registration process actively. This approach is beneficial when human expertise and intuition are crucial in achieving accurate and meaningful alignments.
  2. Semi-Automated: Combining user input and automated algorithms, semi-automated registration strikes a balance between user expertise and computational efficiency. This approach is suitable for tasks where human input enhances the registration process.
  3. Feedback Integration: Incorporating user feedback during the registration process for real-time adjustments, this method ensures continuous refinement based on user input. Real-time adjustments contribute to the accuracy and efficiency of the registration outcome.
  4. Fully Automatic: Requiring minimal to no user intervention, fully automatic registration relies entirely on automated algorithms. This approach is suitable for scenarios where the registration task is well defined and can be efficiently executed without human intervention.

- Classical Methods:
  1. Correlation-based: This method is extensively used for registering monomodal images, playing a crucial role in medical applications. By computing the correlation between images, similarities and discrepancies are identified, allowing for image alignment essential for further analysis and evaluation in medical contexts [12].
  2. Entropy-based: Explores measures of entropy to quantify the information content and patterns within images. Particularly useful in multimodal image registration, this method enhances accuracy by incorporating spatial information into entropy estimation. This innovation aims to provide more reliable results, especially in scenarios where traditional methods may fall short [35].
  3. Mutual information-based: Leveraging pyramid and window-based techniques, mutual information-based methods estimate the probability of comparable voxels in registered images. This facilitates the alignment of images by maximizing the mutual information, offering an effective approach for establishing correspondence between features and achieving accurate alignment [36].
  4. Wavelet-based: This method utilizes wavelet techniques, offering both time and frequency selectivity. Wavelet-based techniques provide a valuable approach for accurate and efficient image registration across different scales and frequencies. The ability to capture information at various resolutions enhances the method’s adaptability to diverse scenarios [37].
  5. Contour-based: Involves matching image feature points using statistical features that describe object contours. With applications in remote sensing, medical imaging, and object recognition, this method leverages the distinctive properties of object boundaries to establish accurate correspondences and align images effectively [38].

- Learning-Based Methods:

Learning-based methods in image registration have gained significant traction in recent years. These approaches leverage machine learning and deep learning techniques to improve registration accuracy, speed, and adaptability across various imaging modalities and applications. The evolution of learning-based methods has been driven by advancements in computational power, the availability of large-scale datasets, and innovations in neural network architectures. Common training approaches include supervised learning, where models learn from paired pre-aligned images; unsupervised learning, which does not require pre-aligned data; and semi-supervised techniques, which combine both approaches [39,40].

These approaches rely heavily on the datasets used for training, which vary widely, depending on the specific application. In medical imaging, datasets may include multimodal brain MRI scans or chest CT images, while remote sensing applications often use satellite imagery. The choice of dataset significantly influences the model performance and generalizability, presenting key challenges such as ensuring robustness in different image qualities and modalities, handling large deformations, and maintaining computational efficiency. Recent research has focused on addressing these challenges through novel network architectures, loss functions, and training strategies, aiming to enhance the versatility and effectiveness of learning-based image registration methods.

In the following, specific approaches within the learning-based methods will be explored:

1. Soft Computing-Based: Integrates artificial neural networks [41], genetic algorithms [42], and fuzzy sets [43] to achieve precise image alignment. This sophisticated approach combines computational intelligence techniques with optimization strategies, resulting in improved accuracy and robustness in image registration outcomes.
2. Curvatures-Based: Another approach in image registration involves curvatures-based methods, which focus on registering a sequence of corresponding points and searching for dimensional projection radiographs. These methods aim to find an optimal fit of the local curvatures in two curves to achieve accurate alignment. This approach is particularly useful in scenarios where the shapes or contours of objects are critical for alignment, such as in medical imaging or object recognition tasks [44].
3. Learning-Based with CNNs: Leveraging convolutional neural networks (CNNs), this method autonomously learns registration patterns and deformable transformations directly from image data. CNNs enhance the accuracy, speed, and adaptability to various types of medical and multimodal images, making them a promising solution in modern image registration [45].
4. Generative Adversarial Networks (GANs): Utilizing GANs, this method generates registered images directly with the deformation field, achieving accurate registration in real time. GANs’ ability to learn and generate realistic images makes them a cutting-edge solution in learning-based image registration [46].

**Figure 6:**
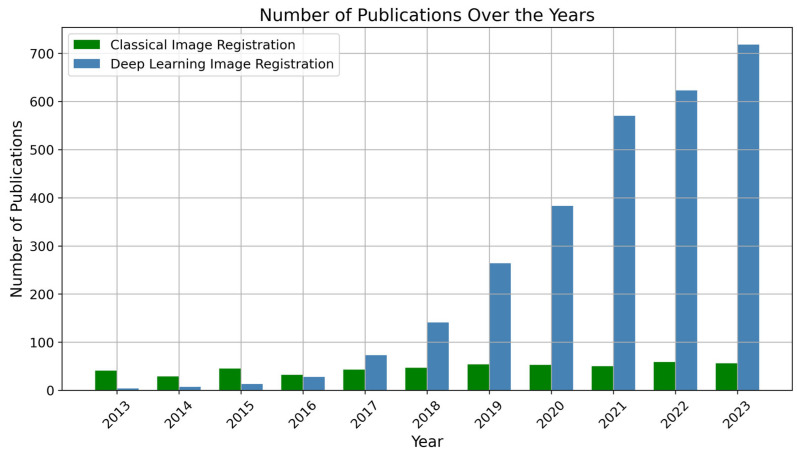
Number of publications in classical image registration versus deep learning image registration in recent years. This plot provides a comparative analysis of the number of publications in classical image registration and deep learning image registration over the past 10 years (2013–2023) [47]. The red line represents the publications in classical image registration, while the green line represents the publications in deep learning image registration. The figure highlights the evolving trends and growing interest in deep learning approaches within the field of image registration, indicating a shift towards leveraging neural networks and machine learning techniques for improved registration performance.

As we delve into learning-based methods for image registration, it is crucial to consider the trends observed in recent research. Figure 6 provides a comparative analysis of the number of publications in classical image registration versus deep learning image registration over the past decade (2013–2023). The plot shows how image registration techniques have evolved over time, particularly with an increased interest in deep learning.

Recent advancements in learning-based methods have explored the integration of attention mechanisms, transformer architectures, and multi-task learning approaches. These innovations aim to improve the model’s ability to focus on relevant image features, handle global and local deformations simultaneously, and leverage information from related tasks to enhance registration performance.

These diverse approaches and ongoing innovations in learning-based methods highlight the dynamic nature of image registration techniques and their integration into the broader landscape of image processing methodologies.

Considering these developments and the various methods discussed throughout this section, we can appreciate the importance of a comprehensive classification framework. In conclusion, the classification of image registration methods presented in this section offers a comprehensive framework for understanding and organizing the diverse approaches in the field. This classification framework not only aids in understanding the existing methodologies but also serves as a roadmap for future advancements, fostering innovation and progress in the dynamic field of image registration.

## 4. Challenges in the Image Registration Process

The process of image registration in the medical field presents a wide variety of challenges that researchers and clinicians must struggle with. One of the greatest challenges is achieving a balance between efficiency, accuracy, and robustness in registration algorithms. The need for registrations to be computationally efficient while still maintaining high levels of accuracy and robustness is crucial for successful image-guided surgeries and diagnostic decision-making.

### 4.1. Technical Challenges in Image Registration

Image registration techniques encounter various technical challenges that affect their application in medical imaging. These challenges include:

- Complexity in Choosing and Implementing Similarity Metrics: Selecting appropriate similarity measures is another critical aspect of the registration process. Various measures, such as mutual information and correlation coefficient, are utilized to evaluate voxel intensity differences between source and target images. However, accurately estimating these measures, especially in the presence of noise and spatial variations, poses significant challenges.
- Outliers and Data Quality: Addressing outliers and rejecting erroneous data points is another ongoing challenge in medical image registration. Outliers can significantly impact the accuracy of registration results, particularly in image-guided surgery applications where an accurate alignment is crucial. Various methods, including robust statistical methods and outlier rejection algorithms, such as RANSAC (Random Sample Consensus), can minimize the impact of outliers. Consistency tests and intensity transformations can also be used to filter out erroneous data points, enhancing the reliability of the registration process. However, achieving robustness against outliers remains an area of active research.
- Optimization Convergence: The convergence of optimization methods to local maxima presents a significant obstacle in the registration process. Optimization techniques play a crucial role in finding the optimal transformation between images, but the risk of converging to the local maxima can compromise registration accuracy. Developing advanced optimization strategies that avoid the local maxima while improving the registration performance is essential for overcoming this challenge and advancing the field of medical image registration [48].
- Geometric Distortion: Geometric distortion, a common issue in remote sensing images, poses a significant challenge in medical imaging as well. Geometric distortion arises from attempts to represent three-dimensional images in two dimensions, leading to relief displacement. Scale distortion and skew distortion are two forms of geometric distortion that can lead to misalignments, complicating the registration process.
- Differences in Image Properties: Differences in images, such as variations in intensity, contrast, resolution, sensor characteristics, and environmental noise, present formidable challenges. Images captured at different wavelengths by diverse sensors and at various times may exhibit discrepancies in resolution, contrast, and illumination, making direct comparisons challenging. This disparity in image characteristics complicates the construction of descriptors that provide global information about feature points, hindering the matching process and contributing to misalignments during registration [49].

### 4.2. Multimodal Registration Challenges

In the field of multimodal image registration, the types of images that can be effectively registered typically include those acquired from imaging modalities that share common anatomical structures and features, despite their inherent differences in imaging principles and characteristics. For instance, MRI and CT images are commonly registered due to their complementary strengths in soft tissue and bone visualization, respectively. Many literature reviews have highlighted successful registrations across various modalities, such as PET-CT for cancer diagnosis, where PET scans provide metabolic information and CT scans offer anatomical context, aiding in accurate tumor localization and treatment planning. Conversely, registering images from vastly different modalities, such as MRI and ultrasound, poses greater challenges due to significant differences in image resolution, artifact patterns, and acquisition principles [50]. Despite advancements, achieving robust registration in such cases remains an active area of research, with hybrid approaches integrating feature-based and intensity-based methods showing promise. Thus, while many combinations of modalities can be successfully registered, the effectiveness often depends on the specific characteristics and alignment challenges inherent to each pair of modalities.

In addressing these challenges, clinical practice often emphasizes focusing on specific regions of interest (ROIs) rather than aligning every pixel within an image. This targeted approach simplifies the registration process and enhances accuracy by directing computational efforts to clinically relevant areas. For example, aligning MRI and CT images may prioritize specific anatomical structures such as tumors or organs critical for image-guided interventions and treatment planning. By concentrating on ROIs, clinicians streamline the registration process and improve alignment precision where it matters most [51].

### 4.3. Interpatient and Intra-Patient Registration Challenges

Interpatient and intra-patient registration in medical imaging face distinct challenges and require specialized solutions. Interpatient registration aims to align images from different individuals, accommodating anatomical variations such as differences in organ shapes, sizes, and positions. Deformable registration techniques are crucial here, capable of modeling complex anatomical transformations influenced by factors like patient movement during scanning and variations in physiological states. Intra-patient registration, on the other hand, involves aligning images acquired from the same patient over time or under different conditions, like varying scan protocols or slice spacing. These differences in acquisition parameters can lead to discrepancies in image appearances, necessitating adaptive registration methodologies. Additionally, the variability introduced by different scan protocols, slice spacing, and scanner manufacturers poses challenges to both interpatient and intra-patient registration processes. Advanced deformable registration algorithms, leveraging anatomical priors or biomechanical models, play a critical role in addressing these challenges. These methods ensure precise alignment essential for longitudinal studies, treatment monitoring, and personalized medicine applications across diverse patient cohorts and imaging setups [52,53].

Advanced deformable registration algorithms leveraging anatomical priors or biomechanical models play a critical role in addressing these challenges. These methods ensure the precise alignment essential for longitudinal studies, treatment monitoring, and personalized medicine applications across diverse patient cohorts and imaging setups. Techniques like these allow for the modeling of complex anatomical transformations and adaptations to variations in image acquisition parameters, leading to more accurate and robust registrations.

Nonetheless, careful consideration must be given when selecting higher-order registration techniques. While these methods have the potential to offer greater accuracy, they also risk introducing distortions if not properly constrained. It is essential to balance the desire for precise registration with the need to preserve the inherent properties of the original images. Therefore, higher-order methods should be evaluated judiciously to ensure they do not alter the fundamental image characteristics in the pursuit of optimal alignment.

In conclusion, the challenges in medical image registration are varied and complex, requiring ongoing innovation and technique improvement. By developing and refining methodologies that account for diverse imaging modalities and patient-specific anatomical variations, researchers and clinicians can enhance the accuracy and reliability of image-based diagnostic and therapeutic interventions.

## 5. Summary

Image registration is a fundamental process with wide-ranging applications, crucial for change detection, target identification, and medical diagnosis. It addresses challenges in aligning images acquired at different times, angles, or devices, offering solutions through multiview, multitemporal, and multimodal analysis techniques.

Medical image registration plays a crucial role in modern healthcare by enabling the fusing of various imaging modalities for the comprehensive analysis and diagnosis of biological structures. With the increasing use of imaging techniques like X-ray, ultrasound, MRI, and CT scans, the demand for accurate and reliable registration methods has grown significantly. This paper provides an in-depth examination of the challenges and techniques involved in medical image registration, aiming to contribute to the advancement of this critical field.

The paper begins by highlighting the importance of medical image registration in integrating diverse imaging modalities for improved diagnosis and treatment planning. It outlines different types of image registration, including unimodal, multimodal, interpatient, and intra-patient registration, emphasizing their significance in various medical applications. The classification of image registration methods based on modalities, transformation types, and interaction levels is discussed, providing insights into the diverse approaches used in this field.

Moreover, the challenges encountered in medical image registration, such as geometric distortion, differences in image properties, outliers, and optimization convergence, are thoroughly examined. Strategies for addressing these challenges, including advanced transformation models, robust similarity measures, and optimization algorithms, are explored. The paper underscores the need for the ongoing innovation and refinement of techniques to enhance the accuracy and reliability of medical image registration systems.

Moving forward, further research is necessary to investigate emerging trends in medical image registration, such as the integration of deep learning techniques, the development of novel similarity measures, and the enhancement of optimization algorithms.
